# Ocular Trauma Score in Siderosis Bulbi With Retained Intraocular Foreign Body

**DOI:** 10.1097/MD.0000000000001533

**Published:** 2015-10-02

**Authors:** Lili Zhu, Pingyu Shen, Hong Lu, Chixin Du, Jianqin Shen, Yangshun Gu

**Affiliations:** From the Department of Ophthalmology, The First Affiliated Hospital, Medical College, Zhejiang University, Hangzhou (ZLL, LH, DCX, SJQ, GYS); and Department of Ophthalmology, The Second Shaoxing Hospital, Shaoxing, China (SPY).

## Abstract

The purpose of this study was to investigate the clinical characteristic and visual outcome of siderosis bulbi with retained intraocular foreign body (IOFB) and to validate the predictive value of the Ocular Trauma Score (OTS) in siderosis bulbi. Certain numerical values rendered to the OTS variables at present were summated (Table 1) and converted into 5 OTS categories as performed in the OTS study. The prognostic value of OTS was first assessed in cases of siderosis bulbi resulting from a chemical reaction of retained IOFBs. Twenty-four eyes of 24 patients diagnosed with siderosis bulbi who underwent surgery between 2007 and 2013 at our medical centre were reviewed. Due to patients’ ignorance in ocular injuries, delayed presentation by the patient (54.17%) and no history of trauma (16.67%) were the most common cause of siderosis bulbi with IOFB retention. The main symptom of all these patients was impaired vision. The most common complications were cataract (23/24, 95.83%), followed by retinal pigmentary degeneration (15/22, 68.18%), iris heterochromia (14/24, 58.33%), pupillary mydriasis (10/21, 47.62%), secondary glaucoma (6/24, 25.00%), relative afferent pupillary defect (6/24, 25.00%), and retinal detachment (3/24, 12.50%). IOFBs were removed in 22 eyes (91.67%), except 2 enucleated eyes with absolute glaucoma (8.33%). Among all the patients (24 eyes), the best-corrected visual acuity improved in 63.64%, unchanged in 18.18% and deteriorated in 18.18% after surgical intervention. No statistically significant difference was found between the categorical distributions of our patients and those in the OTS study group.

Further promotion and education on eye protection are needed to minimize visual loss from siderosis bulbi. The OTS, which was designed to predict visual outcomes of general ocular trauma, may also provide reliable information about the prognosis of siderosis bulbi resulting from a chemical reaction of retained IOFBs.

## INTRODUCTION

Siderosis bulbi is a chronic degenerative process induced by chemical reactions between ocular tissues and iron particles from retained iron-containing intraocular foreign bodies (IOFBs), which may occur from 18 days up to many years. Because of the low incidence and diverse literature data.^1,2^ reliable counseling about siderosis bulbi with retained IOFB remains difficult.

In 2002, Kuhn et al developed the Ocular Trauma Score (OTS), a prognostic model to predict the visual outcome in ocular injuries.^3^ OTS has widely been applied to the assessment of ocular trauma and is highly predictive in various eye injuries.^4–6^ Till now, the prognostic value of OTS for siderosis bulbi remains undefined. The purpose of this study is to assess the prognostic value of OTS in cases of siderosis bulbi due to retained iron-containing IOFBs.

## PATIENTS AND METHODS

The patients, diagnosed with siderosis bulbi between 2007 and 2013, at the Department of Ophthalmology, First Affiliated Hospital of Zhejiang University, China, were identified. The medical records of those patients with surgical removal of the IOFB and a postoperative follow-up period longer than 6 months were reviewed retrospectively.

The demographic data included age, gender, laterality (right, left, or both eyes), injury circumstance, complete medical history, and causes of IOFB retention. Details of each case included injury mechanism, the time interval from injury to IOFB removal, a comprehensive record of the initial ophthalmic examination, entry site and location of IOFB, surgical treatment, and postoperative follow-up. Patients with prior ocular diseases history were excluded, which included corneal disease, cataract, glaucoma, uveitis, vitreous, and retinal problem.

The OTS was calculated for each patient by adding the determined number of variables (visual acuity [VA], rupture, endophthalmitis, perforating or penetrating injury, retinal detachment, and afferent pupillary defect) at presentation (Table 1) and then converted to OTS categories.^3^ The final VA of our study group was compared to those in OTS study group. The categorical comparison was done to assess the likelihood of final VA in the current study and the OTS study.

**TABLE 1:**
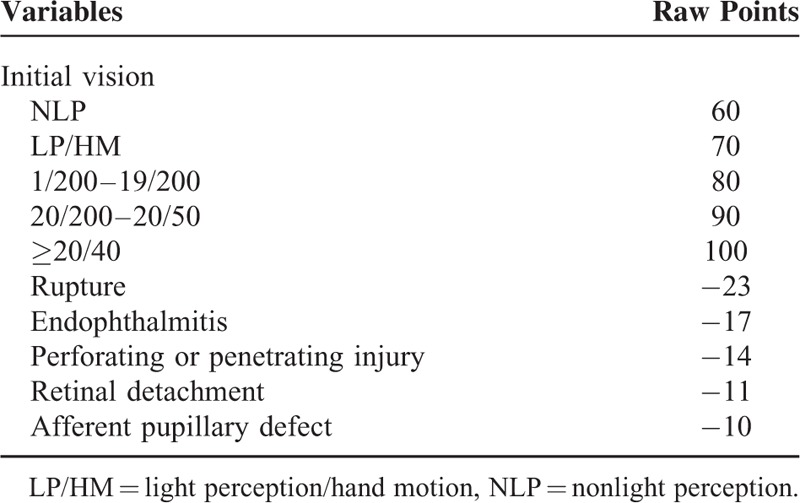
Ocular Trauma Score (OTS)

This study was conducted in accordance with the Declaration of Helsinki, and approved by the ethics committee of The First Affiliated Hospital, Medical College, Zhejiang University, China. Informed consent was obtained from all patients. The Pearson Chi-squared test and Fischer exact test were used for statistical analysis. A *P* value of less than 0.05 was considered statistically significant.

## RESULTS

A total of 24 patients (24 eyes) were enrolled in the study. The average age of the 22 men (91.7%) and 2 women (8.3%) was 39.9 ± 12.2 (standard deviation [SD]) (range 19–67) years. The majority of siderosis bulbi with a history of trauma (18/20, 90.0%) were due to occupational injuries. Right and left eyes were involved in 54.2% and 45.8% of patients, respectively. The mean follow-up time was 14.75 ± 8.68 (SD) months, with a range from 6 to 30 months.

The injuries occurred as a result of hammering in 41.67%, chiseling in 16.67%, lathe turning in 16.67%, electric welding in 4.17%, nail gun in 4.17%, and unknown causes in 16.67% (Table 2). Delayed presentation by patients (54.17%), missed diagnosis or delayed referral (25.00%), no history of trauma (16.67%) and undetected IOFB by computed tomography (CT) (4.17%) prolonged the interval from ocular trauma to IOFB discovery, which led to siderosis bulbi. The period between the ocular injury and the diagnosis of ocular siderosis ranged from 1 to 240 months (mean ± SD: 43.55 ± 68.74 months) in 20 patients (83.33%) with an exact injury history. A total of 17 atients (70.83%) without seeing a doctor or without an exact history of ocular trauma did not receive any antibiotic treatment. No eyes developed infective endophthalmitis in this study.

**TABLE 2:**
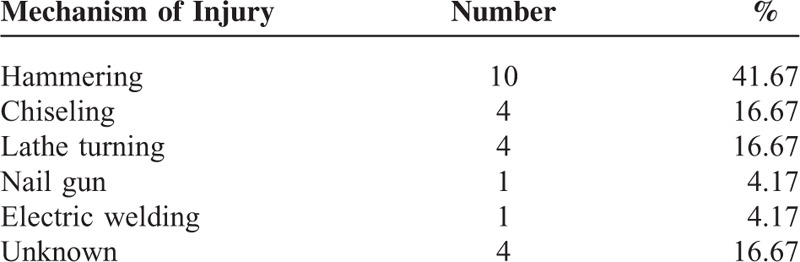
Mechanisms of Injury

The clinical characteristics of the ocular siderosis with retained IOFB are shown in Table 3. The most common complications observed were cataract (23/24, 95.83%), followed by retinal pigmentary degeneration (15/22, 68.18%), iris heterochromia (14/24, 58.33%), pupillary mydriasis (10/21, 47.62%), secondary glaucoma (6/24, 25.00%), relative afferent pupillary defect (6/24, 25.00%), and retinal detachment (3/24, 12.50%). The electroretinogram was depressed in 12 eyes (50.00%) and was normal in 12 eyes (50.00%).

**TABLE 3:**
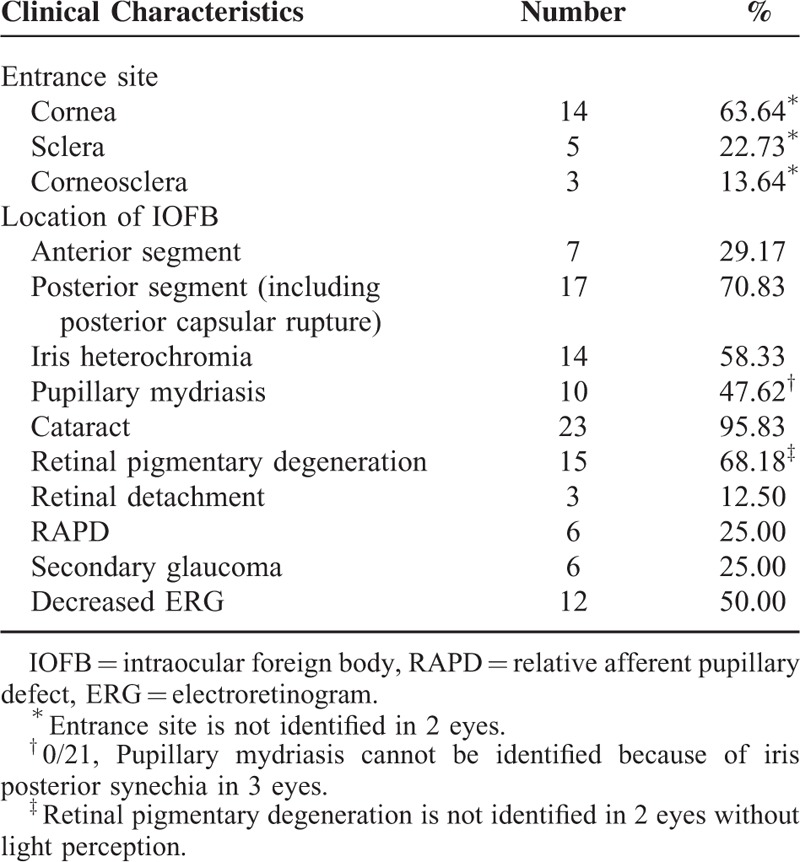
The Clinical Characteristics of Siderosis Bulbi With Retained IOFB

No primary repair was performed at the time of injury. Two of the 24 eyes (8.33%) underwent primary enucleation because of absolute glaucoma with acute pain. Among the remaining cases (22 eyes), IOFBs were all removed via a limbal incision (7/22, 31.82%) or by intraocular forceps with a pars plana vitrectomy (15/22, 68.18%). The lens opacity was extracted by phacoemulsification/lensectomy in 21 eyes (21/22, 95.45%) and the posterior trauma was treated by pars plana vitrectomy (15/22, 68.18%). Intraocular lens were implanted according to the formula SRK-T whenever possible (17/22, 77.27%).

A summary of presenting VA and final VA is shown in Table 4. Initial vision was nonlight perception (NLP, 9.3%), light perception/hand moving (LP/HM, 11.1%), 1/200–19/200 (66.7%), 20/200–20/50 (11.1%), and ≥20/40 (1.9%). At the last follow-up, the best-corrected VA was NLP (9.3%), LP/HM (11.1%), 1/200–19/200 (11.1%), 20/200–20/50 (53.7%), and ≥20/40 (14.8%). Compared with the initial VA, the final VA improved in 58.33% of cases, remained unchanged in 25.00% of cases, and deteriorated in 16.67% of cases, in whom all IOFB passed to posterior segment. Final VA in the OTS categories of our study and those in Kuhn et al's OTS study group are presented in Table 5. Comparing the categorical distribution in our study with those in the OTS study, all of the *P* values were greater than 0.05 (Table 5).

**TABLE 4:**
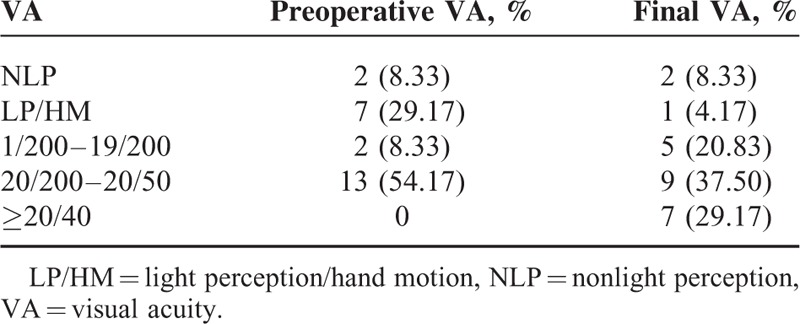
Preoperative and Final VA

**TABLE 5:**
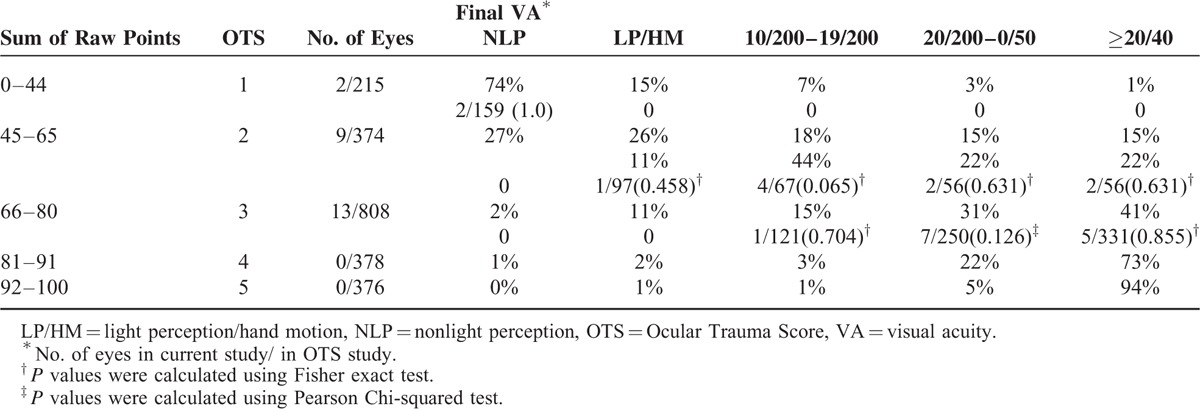
Calculating the OTS: Conversion of Raw Points into an OTS Category, and Calculating the Likelihood of the Final Visual Acuity in 5 Categories

## DISCUSSION

Siderosis bulbi is 1 sight-threatening complication that is triggered by iron-containing IOFB. Penetrating injuries involving retained IOFBs which are almost ferromagnetic represent a significant subset of ocular injuries.^7^ Similar to other studies,^8–10^ males constituted 91.7% of the patients, with a male-to-female ratio of 11 to 1. This may likely be due to the high proportion of occupational injuries (18/20, 90%) from dangerous jobs in which mostly have a higher percentage of men. This study also revealed that hammering was the most common mechanism of injury, which is similar to other studies.^11,12^

The average length of time that elapsed between ocular injuries and the discovery of siderosis was 43.55 months (range 1–240 months) in those cases whose history of injury could be elicited. Delayed presentation by the patient (54.17%) and no history of trauma (16.67%) prolonged the interval from trauma to the diagnosis of siderosis bulbi. Most patients (83.33%) in this study were blue collar workers (physical laborers) who often lack self-protection awareness. All these patients did not use proper eye protection when conducting hazardous tasks. Poverty and deficiency in self-health awareness hampered the timely treatment for most patients in our study. Siderosis bulbi in this tertiary referral hospital was partly due to late-delivered IOFB, which was neither suspected nor examined carefully in the local hospitals (25.00%). The small IOFB in 1 patient (4.17%) was missed by CT, although CT is considered as a gold standard for IOFB detection. The reason may be that the diameter of tiny foreign body is smaller than CT slice thickness.^13^

Due to its low incidence, most articles on siderosis bulbi are case reports describing clinical signs. Clinical findings in siderosis bulbi include iris heterochromia, pupillary mydriasis, cataract formation, retinal pigmentary degeneration, and secondary glaucoma. This study showed that the complications observed were cataract in 23 (23/24, 95.83%) patients, retinal pigmentary degeneration in 15 (15/22, 68.18%) patients, iris heterochromia in 14 (14/24, 58.33%) patients, pupillary mydriasis in 10 (10/21,47.62%) patients, secondary glaucoma in 6 (6/24, 25.00%) patients, relative afferent pupillary defect in 6 (6/24, 25.00%) patients, and retinal detachment in 3 (3/24, 12.50%) patients. Although the characteristics of siderosis bulbi have been described in many studies.^14–16^ The prediction of visual outcome in siderosis bulbi are poorly known and not well studied. Because of its sophisticated clinical characteristics, it is difficult to inform the patient and family about the visual prognosis of siderosis bulbi with retained IOFBs.

The removal of IOFB can stop the progression of siderosis,^17^ and our goal is to discover and remove the IOFB in cases of siderosis bulbi without delay. In spite of a conservative approach adopted in cases of small, local bodies with quiet eyes.^18^ Early surgical removal of the metallic IOFB should be recommended with recent surgical advances that enable safe removal of the IOFB with good visual result. This finding suggests that the surgical treatment can improve the visual rehabilitation in most of siderosis bulbi cases (14/22, 63.64%). With respect to the deteriorated visual outcome, a posterior IOFB next to the macular is a risk factor that may cause macular epiretinal membrane formation and scar in the fovea. It is easily explainable considering that the development of macular lesion is associated with worse visual prognosis.

OTS has been validated as an effective and precise tool for predicting visual outcome in many kinds of mechanical eye injuries including penetrating injuries with IOFB.^19–21^ However, to the best of our knowledge, the prognostic value of OTS has not been evaluated in siderosis bulbi as a result of a chemical reaction from retained ferritic IOFB. Our study showed that the likelihood of the visual outcomes in our case series was similar to those in the OTS study. The OTS model may provide reliable predictive information about the visual outcomes of siderosis bulbi after our surgical intervention. Higher OTS categories tend to indicate a better prognosis and remain the most important prognostic factors when counseling the patient and his family.

Prevention might minimize the sight-threating effect of siderosis bulbi that is preventable. Higher public awareness of protection against ocular injuries related to dangerous work should be strengthened,^22^ and appropriate measures for eye protection should be widely encouraged.^23^ Increasing awareness of the serious nature of siderosis bulbi with retained IOFB would help educate both blue-collar workers and medical professionals in the primary hospital. Based on the heterogeneity of clinical findings, prompt and full assessment of patients with possible IOFB retention is important to provide timely delivery or appropriate management and to improve the final visual prognosis.

The results of this study should be considered in light of several limitations. This study could not assess the clinical signs associated with visual prognosis because of a small case series. There may be a geographical bias that is inherent to most ophthalmic trauma studies. Possible treatment selection bias may have affected our results. The limited power of the study is because of the intrinsic shortcomings of such retrospective reviews. However, these limitations do not significantly affect the major findings of this study.

In conclusion, our study showed that the primary victims in siderosis bulbi were young male who suffered from work-related injuries. Of the most patients with surgical management, the best-corrected VA at last follow-up was better than initial VA. In siderosis bulbi, OTS can offer the possibility of simple approximation of the functional result, without significant difference between the categorical distributions of this study patients and those in the OTS group. Further education on ocular injuries for physical laborers and ophthalmologists in primary hospital is recommended.
